# From National Biosecurity Measures to Territorial ASF Preparedness: The Case of Free-Range Pig Farming in Corsica, France

**DOI:** 10.3389/fvets.2021.689163

**Published:** 2021-07-28

**Authors:** Marie Gisclard, François Charrier, Bastien Trabucco, François Casabianca

**Affiliations:** ^1^SELMET-LRDE, INRAE, ACT Department, Corte, France; ^2^LISIS, INRAE, ACT Department, Marne-la-Vallée, France; ^3^INRAE, Centre de Corse, San Giuliano, France

**Keywords:** African swine fever, Corsica, free-ranging farming system, territorial preparedness, biosecurity, social acceptability, outdoor farming system

## Abstract

In response to African swine fever (ASF) outbreaks in wild boars in Belgium in 2018, the French authorities issued national biosecurity measures for all pig farms, regardless of their geographical and socio-technical scale. Considering the Corsican pig farmers' demonstrations against these measures (for geographical, cultural, and economic reasons), this article questions the suitability of standardized top-down national measures that potentially endanger traditional breeding systems, which are increasingly marginalized in relation to the dominant industrial model. From an action-research approach, the article analyzes how local stakeholders go beyond usual classical biosecurity issues to propose a territorialized preparedness. Mediating between Corsican farmers and the government representatives, a technical committee made up of actors from various regional research and development bodies drew up a socially acceptable preparedness proposal. Viewing the health risk from a local standpoint, the committee provided arguments for maintaining the extensive grazing that is non-negotiable for the farmers, while getting the farmers to agree to change other practices (reproduction control) as a measure against health hazards already present. Analysis of the preparedness process and the mediation process shows that a territorialized bottom-up approach to the governance of health risks can make biosecurity measures more acceptable to farmers. It also points to the legitimacy of a set of alternatives to top-down measures that standardize farming systems and may lead to the disappearance of small farmers and their traditional systems.

## Introduction

African swine fever (ASF) is a highly contagious viral disease specific to wild and domestic swine, with no danger to humans but with serious consequences for animal health. France has been free of ASF since 1974 (1), but the virus has been endemic since 1978 in Sardinia, an island only 12 km from the coast of Corsica. It entered Europe in 2007 through the Caucasus and has spread throughout Eastern Europe and Asia, where it threatens the pig industry in affected countries. In 2018, ASF was detected in Belgium (2). Shortly afterward, the French government issued a Ministerial Decree (3) prescribing biosecurity measures for all pig farms, regardless of their location and socio-technical characteristics. In the case of pig farming, biosecurity measures are designed to limit interactions with wildlife and with other farms by installing fences or confining the pigs. So, the decree includes strong measures to set up double enclosures and to fence all outdoor farms with grazing land.

Preparedness as “*a style of reasoning and a set of governmental techniques for approaching uncertain threats*” (4) and biosecurity, which has become a major pillar of preparedness for emerging infectious diseases [swine flu, ASF, severe acute respiratory syndrome (SARS), etc.], are often standardized (5, 6) despite the diversity of local conditions and farming practices (7). There can be wide discrepancies between biosecurity techniques and the technical characteristics of farming systems and between a national preparedness plan and a potentially wide range of local issues, which can go beyond disease management (8). Some studies show that national preparedness measures are out of step with local issues and situations (9, 10). Also, health risk is rarely considered from a territorial perspective (11). All these contribute to the global standardization of pig farming systems and the dissemination of the industrial farming model throughout the world (12, 13).

As a consequence, biosecurity plans and national preparedness may be rejected by livestock farmers and other health stakeholders (14) because of the diversity of contexts in which livestock disease outbreaks arise (15, 16). The decontextualized nature of classical biosecurity measures therefore constitutes a first obstacle for the design of an effective, applicable preparedness plan in a given local territory.

In fact, pig farmers on the French Mediterranean island of Corsica mobilized to contest the implementation of the national decree. They consider the control measures as unapplicable because of the predominant free-ranging farming systems, the mountain topography and land tenure issues. Indeed, classical biosecurity measures are much harder to implement when pigs have access to pastures shared by different herds (17) or with wild boars nearby. Corsica's pig farming systems have been considered unconventional (18) in comparison to both indoor and outdoor pig farming systems in mainland France. Corsica's pigs are destined for dry-cured meat production, processed and retailed by the farmer him/herself, with small herds averaging 90 to 200 pigs slaughtered per year (19). Huge areas of unfenced pastureland are vital to these systems, as they provide the chestnuts and acorns that are key to the pigs' diet and are mandatory stipulated in the Protected Designation of Origin (PDO) specifications (20). The pasturelands are thus a significant resource for the Corsican pig sector's development and the typicality and renown of its products. They are also a source of public subsidies for their contribution to countryside maintenance. So, the announcement of the new national biosecurity obligations raised major cultural and economic issues and made farmers fear the disappearance of their traditional farming systems.

However, from an epidemiological point of view, Corsica is a vulnerable territory because of its geography and the interaction between livestock and wild boars on the unfenced pasturelands (19, 21, 22). The epidemiological situation may be considered worrying, as the Aujeszky virus is circulating at a high rate (23) and bovine tuberculosis is reemerging (24). Epidemiological surveillance and management are complicated by the presence of informal farming and clandestine on-farm slaughtering. So, it seems very difficult to implement national biosecurity measures against African swine fever virus (ASFV) introduction but unrealistic to maintain the status quo. Following farmers' protests, several research and development organizations got together to form a technical committee (TC). The TC evaluated the overall situation as an opportunity to address the weaknesses of health management in the Corsican pig sector.

The notion of acceptability (8, 25) allows to understand the potential gap between management measures based on official expert risk assessments and the implementation of those measures and the social conflicts that arise (15). The notion of acceptability points to a dynamic process (26) through which a compromise can emerge and stabilize. It is achieved through important phases of contestation, deliberation, and negotiation to reach a compromise between administrators and citizens of the territories concerned (27, 28). The construction of compromise is “intermediated” through the emergence of various actors or groups of actors (consumers, farmers, associations, etc.) who coordinate to achieve change (29). Looking at mediation as a way of building compromise required us to particularly analyze the actor legitimacy, the stability of local collectives, and the ability of local actors to carry the process through.

Classical biosecurity measures against ASF call into question the existence and legitimacy of small farms that use pasturelands classified as at-risk. French outdoor pig farmers have already negotiated marginal adjustments, including the possibility of penning animals behind fences rather than walls. This is a perfect illustration of the fact that acceptability tests are often carried out by a statistically marginal minority and/or concern some aspect of the project that only affects “marginal” actors (28).

However, Corsican free-range farmers cannot be considered a statistically marginal minority. Although marginal in terms of the French pork sector (<1% of national production), they nevertheless represent the vast majority of the 350 (30) island's pig farmers, whose farming systems are almost unique to Corsica and are only marginal in relation to the rest of France. So, the question of acceptability is raised not at the level of individual farmers but concerns a whole territory.

The uniqueness of Corsica therefore puts to the test a “prescribed” global or universal (14) standardizing approach to biosecurity. Prescription alone cannot work in Corsica without risking serious social, economic, and land management consequences. The traditional system would be doomed to disappear, evolve into a system similar to “outdoor” systems found on the mainland, or go underground. But while the acceptability of biosecurity measures imposing mandatory confinement of animals seems complicated for Corsican farmers, the acceptability of negotiating this central point of the national measures is also not obvious: biosecurity concerns the management of a diffuse risk, in this case, a Category 1 disease whose management is the responsibility of the state. Animal health also has implications for public health (risk of zoonotic diseases, though not in the case of ASF), the agri-food economy, etc.

The aim of this paper is to analyze how the stakeholders of a subnational territory with small farmers practicing free-range livestock systems that deviate sharply from the top-down public policy standard design and negotiate adaptations to its specific features.

Our hypothesis is based on two assumptions:

1. The acceptability of the adaptations cannot be limited to marginal adjustments but must involve building a genuine preparedness that meets the challenges of the local farming system.
2. Local preparedness is a complex organizational process involving different acceptability tests by farmers and the authorities, which will be easier to achieve with someone to mediate between the two groups.

## Materials and Methods

This paper is the result of an action research approach that used qualitative methods: participant observation and semi-structured interviews. In action research, the key element to be analyzed and interpreted is the various collective processes triggered by the researchers' practical involvement alongside other actors seeking change. It follows from the idea of making complex mechanisms (especially social mechanisms) visible and analyzable through real-world intervention (31–33).

### Empirical Data: Participatory Observation and Intervention Research in the Corsican Pig Sector

The first type of material collected was essentially empirical and came from participant observation carried out by the authors, who were members of the TC. As such, they first attended the first two farmers' meetings, at which the farmers formed a collective (the farmers' collective) to protest against the unacceptability of the national ASF biosecurity measures.

Following the creation of this farmers' collective, the TC included multidisciplinary Corsican stakeholders concerned with animal health: the Groupement de Défense Sanitaire (GDS–farmers' association for livestock health protection), the Groupement Technique Vétérinaire (GTV–regional association of veterinarians), the Chambers of Agriculture of the two districts of Corsica, the Regional Chamber of Agriculture (that covers all of Corsica), INRAE, the two departmental hunting federations, the Corsican Office of Agricultural and Rural Development (ODARC), and representatives of the main farmers' organizations. Its aims were (i) to preserve Corsican pig farming by proposing adaptations of the national biosecurity measures and (ii) to improve the health management of pig farming in Corsica, where several pig diseases are already present, by building a Regional Health Plan.

The meetings that are part of our material and have therefore been analyzed concern:

- The farmers' collective meetings (2). The farmers' positions were reported to and discussed by TC members at TC meetings.
- All the TC meetings (17)
- The meeting where the TC presented its work to the pig farmers' collective, at which the farmers adopted the proposed plan and agreed to formally submit it to the authorities
- The three meetings between the TC and the authorities. The first was to make sure the authorities would be willing to consider alternative proposals. The second was for the TC, accompanied by representatives of the farmers' collective, to present its proposals. At the third, government experts gave their opinions on the acceptability (to the authorities) of the proposals put forward, asked for clarifications, and launched a series of actions to obtain agreement from the Ministry of Agriculture. Once the proposals were submitted to the decentralized state services, the latter conducted the negotiations with the Ministry of Agriculture at the national level (specifically, the General Inspector of Veterinary Public Health).

**Figure 1:**
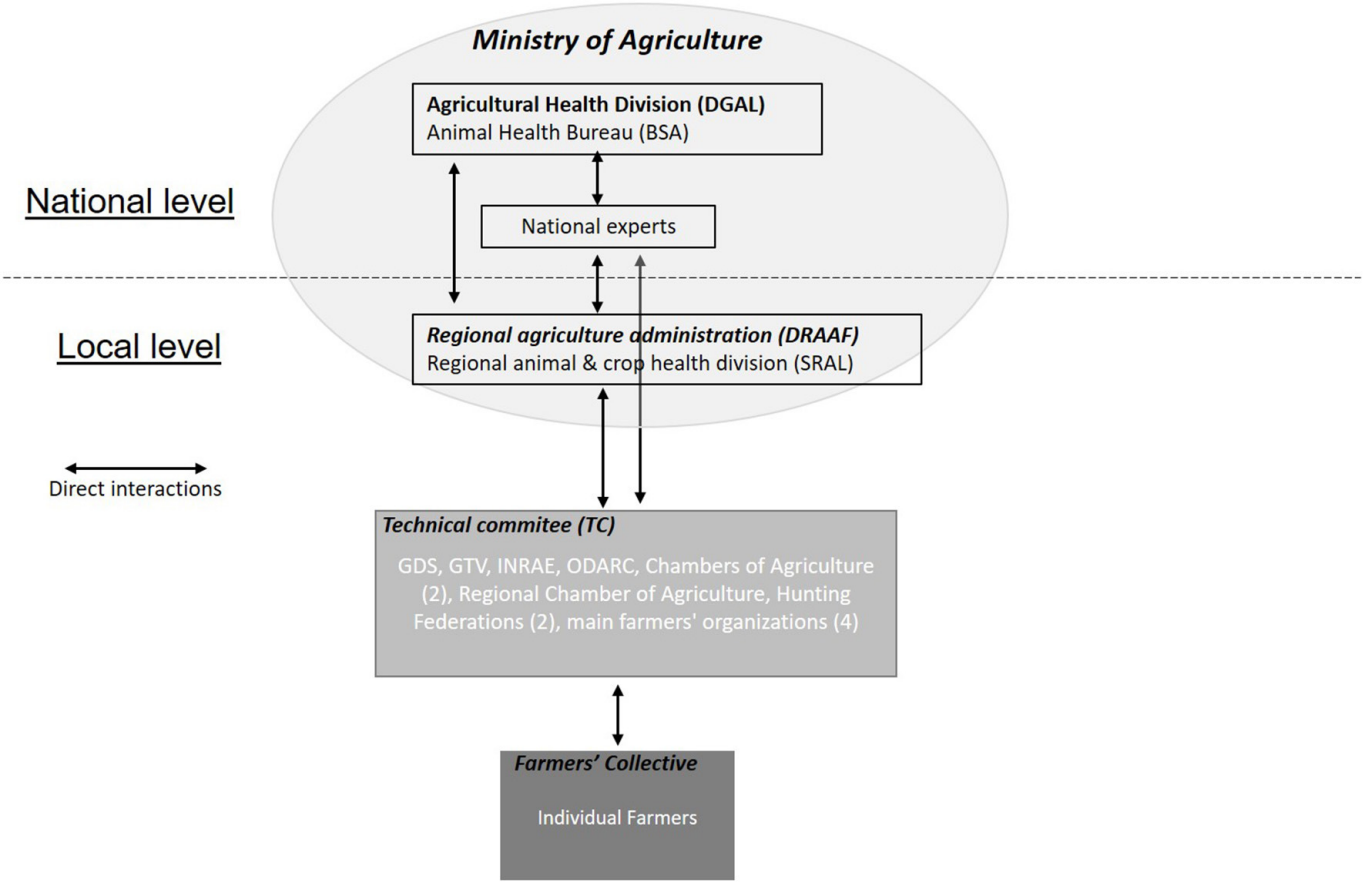
Interactions between the different actors in the territorial preparedness process against African swine fever (ASF) in Corsica (2019–2021).

All these meetings, which were moments of construction, discussion, and negotiation between stakeholders (Figure 1), are listed in Table 1. A report was prepared after each meeting so that the progress of the preparedness process and the negotiations could be monitored and analyzed.

**Table 1:** Number and purpose of the meetings conducted in the territorial preparedness process against ASF in Corsica (2019–2021).

**Meetings**	**Number of meetings**	**Period**	**Purpose of the meeting**
Pig farmers	2	April and June 2019	Mobilization to contest the ministerial decree
TC	17	From July 2019 to September 2020	Building a preparedness process proposing alternatives to national biosecurity measures
Breeders and TC	1	September 2020	Validation of TC proposals by farmers
TC and decentralized state services	2	September 2019 and November 2020	Validation of the alternative proposal process Validation of a preparedness approach
TC, decentralized state services, national experts	1	January 2021	Discussion of the proposals with the experts, identification of sensitive points to work on, and validation of the proposals as a whole
Decentralized State services and national state services (Agriculture Ministry)	2	February and March 2021	Negotiation for government's acceptance of the preparedness proposal

*ASF, African swine fever; TC, technical committee*.

### Semi-Structured Interviews

To supplement the material obtained by participant observation, we then conducted comprehensive semi-structured interviews (34, 35) to shed more light on the different actors' positions as they appeared to us in the various meetings and to gain a better understanding of what was acceptable or unacceptable to them (Table 2). We held these interviews with (i) approximately 30 pig farmers; (ii) two government officials responsible for animal health issues at the regional level about the organization of health management and health surveillance capacity on pig farms; and (iii) five veterinarians, on their relations with farmers (36). The sample of farmers (that represents about 10% of the pig farmers) was selected to be representative of the diversity of Corsican farming systems, classed into four groups according to their level of risk for the introduction of emerging diseases (19). The face-to-face interviews focused on their husbandry practices and the impact of these on pig health in general, their perceptions of the risk of spreading diseases such as ASF, and their knowledge of unsafe practices. The interviews lasted 2 h on average. They were recorded, transcribed in full, and analyzed thematically.

**Table 2:** Farmers' positions at the first two meetings of the pig farmers' collective mobilized against the French national biosecurity measures to fight African swine fever.

**Farmers' positions**	**Problematic/unacceptable elements in the decree**	**Proposals for solving the problem**	**Subject of negotiation**	**“Acceptable” proposal**
“Warrior” (ready to fight the state)	Lack of control of grazing land to be fenced Authoritarian behavior of the state	Preserving the pasturelands Closing Corsica's borders to meat imports Arm wrestling with the state	No negotiation possible	Status quo
“Passive” (resistant to any change)	Not treating insularity as a means of protection from the outside world Ignoring the precedence of husbandry practices presented as ancient and traditional	Closing Corsica's borders to meat imports	Maintaining and improving current border controls	Better state control of illegal imports and increased tourist awareness
“Fatalist” (anyway the authorities make the decisions)	Everything is acceptable, since, in any case, the authorities will do what they have already decided.	Waiting for the obligations to come into effect	Nothing to negotiate	Compliance with the ministerial decree
“Pro-active” (finding suitable solutions)	The end of open upland grazing and therefore of an ancestral activity. PDO specifications would be impossible to meet. Lack of control over land that would have to be fenced. The authoritarian, top-down approach. Too-short deadlines	Closure of Corsica's borders to meat import Designing a gradual plan that would leave no one behind	Assessing the state's ability to control livestock health	Structuring farms Opening a dialogue between organizations and public authorities
“Demanding” (resources for making changes)	Cost of structuring the farm Intolerable financial cost and technical difficulty of fencing pastureland	To free up exceptional resources	Public funding for restructuring the farms Formal commitments of funding authorities	Creating a budget for public funding Compensation for dead animals

### Framework for Analyzing Acceptability Testing

We observed the work of the TC, which was aimed at making proposals acceptable to both the farmers and the government. We analyzed the different stages of the acceptability testing for both the national measures and the TC's proposals. We also analyzed the issues at stake at the time of the testing.

- We first identified the diversity of actors concerned and their positions with regard to the national biosafety measures and the adaptation proposals. We constructed a thematic analysis (37, 38) that makes explicit the standpoint of each stakeholder group toward the application of the national biosecurity measures in Corsica in terms of “problems,” “objects of negotiation,” and “acceptable solutions.” We listed (i) what was problematic for the farmers in the biosecurity measures, (ii) what the authorities saw as problematic in the Corsican pig farming system, and (iii) what the stakeholder groups envisaged doing based on their interpretation of the problem. This highlighted the diversity of positions within each party (the farmers and the authorities) and enabled us to identify the objectives and problems that were critical for each party and which the negotiations were to address.
- Next, we assessed the acceptability testing of the TC proposals using the criteria suggested by Barbier and Nadaï (2015): (i) they make sense to everyone, (ii) they are robust (no other preferable alternatives), (iii) they have safeguards in the event of interference with inappropriate behavior, and (iv) they take into account the diversity of interests and values concerned. We examined how they constituted responses to the elements considered unacceptable by each of the parties. To do this, we compared the TC's solutions with the factors the various stakeholders considered problematic (Tables 3, 4). Validation of the various elements at successive meetings (TC meeting, then meeting between the TC and the farmers' organizations, and finally meeting between the TC and the authorities) was considered in each case a successful step in the acceptability test.

**Table 3:** Proposals from the local technical committee on the unacceptable elements for Corsican pig farmers of the national biosecurity measures against African swine fever.

**Problematic/unacceptable elements in the decree**	**Suggestions for solving the problem**	**“Acceptable” proposal**
Lack of measures specific to island territory	Insert a component specifically designed to protect against the introduction of new health hazards	Effective implementation of measures at ports and airports Raising awareness among importing deli producers Communication with tourists and visitors
Health management problems with many health hazards already present	Local preparedness involving farmers, state, vets, hunters, slaughterhouses	Redistribution of responsibilities between all health actors
Difficulty of building barriers	Realistic, effective changes to herd management Feasibility, gradual change	Maintaining the pasturelands by preventing sexual interaction between wild boar and domestic animals Keep breeding animals penned and accept the risk of deli meat loss Adapt the biosecurity measures to Corsican farms (cancel inappropriate standards, add appropriate, effective elements) Gradually upgrade farms according to their initial situation

**Table 4:** Positions of local and national authorities in the regional health plan negotiations in the territorial preparedness process against ASF in Corsica (2019–2021).

**Negotiating actors**		**Problematic/unacceptable elements**	**Proposals for solving the problem**	**Subject of negotiation**	**“Acceptable” proposal**
State	Regional state	Risk of ASF introduction and dissemination to French pig farms Representative professional organizations	Facilitation to help the sector implement the Regional Health Plan	Corsica's island status	The health status of Corsican farms The risk of the spread of ASF in case of introduction The lack of animal identification
	National state representative in the region (Prefect)	Risk of dissemination to the mainland	Role of the Conseil Régional d'Orientation de la Politique Sanitaire Animale et Végétale (Regional Council for the Orientation of Animal and Plant Health Policy)	Health situation of the island	Not spreading a virus to the continent
	National state (central government)	Health hazards present	Treating bovine tuberculosis (zoonosis)	Specific measures to be activated as soon as possible	Manage the timescale of the various measures of the Community Plan
Regional Community	Agricultural and Rural Development Office	Lack of structure (farm structure and physical structures)	Conditions for aid Nature of reciprocal commitments	Allocation of funds	Creating rules for eligibility of individual applications

*ASF, African swine fever*.

- Lastly, to analyze the role of the TC's mediation work in the acceptability testing process, we established a “storyline” of the preparedness process. The resulting chronicle captures the essential moments in the process of designing and negotiating acceptability and the combinations of actors and arguments that comprised those moments. It shows the temporal sequences and specific focuses (39) that led to the Regional Swine Health Plan. The legitimacy of the TC and its ability to conduct the mediation process were assessed in terms of the continuity and regular timing of the meetings it organized and the discussions it fostered between different stakeholders. The success of the mediation and the stabilization of the negotiation process were judged by the establishment of compromises between stakeholders, giving rise each time to a new stage in the negotiation process.

## Results

Our results show three steps in the acceptability process: (i) a collective acknowledgment of the unacceptability of the national biosecurity measures, (ii) a collective design process aimed at proposing a territorial preparedness appropriate to Corsica's farming systems and addressing the elements considered acceptable or unacceptable by different actors, (iii) and a negotiation dynamic between a diversity of entities. For each step, we highlight the TC's role as mediator for the acceptability of the Regional Health Plan, with the farmers on one hand and the government authorities on the other.

### Arguing the Unacceptability of the National Measures

When the national biosecurity measures were announced, the farmers' collective expressed their rejection of these measures. The reasons are many: Corsica's rugged terrain, the use of large areas of pastureland, and lack of control over these vast expanses, which, furthermore, overlap between private and common land. On the other hand, the “traditional” nature of Corsican farming systems would be undermined by penning the animals behind fences. They see the injunction to confine their animals to protect them as a top-down imposition and a denial of local farming practices.

The concept of “pastureland” cannot be reduced to “outdoor” pig keeping (in fenced pens). Pastureland grazing is the main criterion that differentiates Corsica's pig system and its products. Its disappearance is considered non-negotiable by some, especially those farmers registered in the PDO, whose specifications require the pigs to be grazed on pastureland.

At these meetings of the farmers' collective and in the semi-structured interviews, we identified various positions among the farmers. Table 2 includes five different positions and details the main characteristics of each.

Between the meetings of the farmers' collective, we observed quite noticeable changes in the positions of those in attendance. First of all, while the “warriors” took an uncompromising position against the government at the first meeting, they seemed to disappear, or spoke much less, at the second: Had they understood that most farmers are not sufficiently motivated to rebel against the authorities? Similarly, the “passive” and the “fatalists,” although present at both meetings, appeared less numerous at the second: Were they already tempted to withdraw and join the informal sector? At this second meeting, the “demanding” and “pro-active” who expressed the desire to make proposals to negotiate for appropriate ways and means to meet the health challenge were mostly still present and responsive.

One particular point of tension is felt in the discussions: some farmers (mainly the “passive” ones) think that being an island is in itself a complete solution for protecting Corsica and avoiding the need to restructure the farms. In fact, the lack of infection despite the weakness of the special protective measures between Corsica and Sardinia has lessened farmers' fears. Many feel that the government biosecurity solutions are out of proportion to the danger (still remote and without apparent urgency). The farmers see border control as the main measure to be implemented. The discussions brought to light a practice of purchasing live animals or pork meat from outside the island and mixing local and imported raw materials in “farmhouse” products (that are supposed to consist only of raw materials from animals born and raised on the farm). This opportunistic behavior by some farmers was denounced as a collective risk with regard to the virus. The provisional conclusion from these discussions was that using insularity cannot be considered sufficient: it is necessary to restructure pig farms to make them less vulnerable, especially to the many health hazards already present on the island.

Some farmers agreed that while the biosecurity measures imposed are not acceptable, doing nothing is not acceptable either and certainly will not be accepted by the authorities. There is also the risk of prompting the state to take an authoritarian attitude that would drive the majority of farms into the informal economy. Preparing against ASF means first knowing how to combat the health hazards already present.

This first phase led the farmers' collective to agree on the unacceptability of national measures, but also to three conclusions:

(i) preparation against ASF is an opportunity to tackle the sector's health issues more generally;
(ii) farmers are not alone in having to make efforts (as indicated by the issue of border controls, the state must also be able to protect the island), and it will be necessary to organize a sharing of responsibilities and the mobilization of adequate resources; and
(iii) the need for a TC composed of regional research and development actors to draw up practical proposals to address the local situation.

### Building Locally Acceptable Responses: Toward Territorial Negotiation

The TC first aimed to bring together people working in scientific and technical support for the pig sector. Its composition includes representatives of the hunters (because of the need for wildlife surveillance), and it was suggested that the representatives of the slaughterhouses should also be integrated. One question remained: what should be the place for the professional representatives of the farmers' organizations? How to manage representativeness and balances within the TC? With an *ad hoc* body created from scratch, special care must be taken in establishing its operating rules, with a pre-agreed agenda and minutes taken down by members collectively appointed as secretaries, taking turns. In order to structure the work, the TC drew up a schedule to identify the issues to address over the course of the meetings.

First, the TC carried out a systemic analysis of the situation. Thus, the TC extended its thinking beyond ASF to the health hazards already present as a real emergency. In particular, bovine tuberculosis, which affects some pig farms, appears to be particularly relevant. Designing a regional health plan therefore means including the danger posed by ASF along with the diseases to be considered. As far as flows are concerned, waste management is a very sensitive point and a very effective dissemination route in the current situation. Hunting (abandoned remains) and farm processing (deli waste) are the greatest risks of health hazards in the wild. Major efforts will have to be made to limit these risks.

The TC met mainly in plenary sessions, but at a certain stage, it proved necessary to set up a special “Farm Biosecurity” group in order to adapt the biosecurity measures to the Corsica free-range farming. Several meetings (in working groups and then at a plenary meeting) enabled us to compare points of view and to make proposals that balanced protection of the animals with maintenance of extensive grazing on pastureland. From the hierarchy of risks expressed by farmers and incorporated in its work, the TC identified the main line of its proposals: limiting direct interaction between animals by reproductive control. The sow in heat attracts males from a wide area, leading to intense interaction. This initial reasoning logically led to a set of provisions such as mating confinement and oophorectomy of non-breeding females. This reasoning drew on earlier discussions within the local Nustrale breed selection scheme, a networked genetic management arrangement among pig farmers. Many of the pig farmers already perform oophorectomy on their sows (23). The extra cost to those farmers who do not do it should be paid by ODARC, the Corsican Office of Agricultural and Rural Development (one member of the TC is on the staff of this body), with which the funding of the Regional Health Plan had to be negotiated (Text Box 1).

Box 1 **Main measures of the regional health plan negotiated between pig farmers, local stakeholders, and national authorities in the territorial preparedness process against African swine fever in Corsica (2019–2021).**

The regional health plan is based on three objectives: (1) preventing the disease from entering the territory, (2) detecting its arrival early so as to reduce its impact, and (3) managing actions for its eradication.

First, to achieve these objectives, the TC identified 12 sub-goals and 40 actions and listed the actors responsible for implementing each action. Some of the main actions identified are as follows:

- Improving identification of animals and farmers

- Raising the awareness of farmers and hunters to the issue of managing the waste from hunting and butchery

- Raising farmers' awareness of the importance of using the abattoirs for slaughtering

- Making sure the abattoirs have the capacity to meet all the island's slaughtering needs

- Improving border controls and tourist awareness of the issue

- Setting up experimental management plans for Aujeszky's disease and tuberculosis

The TC then proposed adaptations of the national biosafety measures, involving the following:

- Keeping breeding animals behind double fences

- Using grazing land for castrated animals only to avoid sexual interaction with wild pigs and animals from other farms

- Returning leader sows to the farm as soon as possible after their quarantine

- Creating a quarantine zone on the pastureland in the event of a health danger

- Bringing farms up to standard in staggered order, to allow time for farms that are lagging behind to adapt gradually

The TC then conducted checks along the way with a small number of diverse farmers: would they agree to guard against direct sexual interactions by protecting their sow units? Most of these small farmers both farrow and fatten, with self-replacement of the sows. They confirmed, whatever their position at the start of the debates, that sows and boars represent their basic genetic heritage and that protecting them as operating capital will enable them to continue their business even if there is disease in the environment. But they accepted the possible risk of indirect contact with wildlife or between herds on the pasturelands. Farmers also argued that pregnant sows should continue to have access to the pasturelands. In particular, “leader sows” (older females), followed by their offspring, play a major role in animal-to-animal learning (knowledge of feeding, watering, and sleeping areas) and in managing the herd. The TC then designed a procedure to allow pregnant sows to remain on the pasturelands, returning to the protected breeding unit for a quarantine period before giving birth.

However, increasing the level of reproductive control and getting equipped with the necessary breeding arrangements will require training, time, and resources. The TC then held thorough-going discussions on the changes that would allow for the diversity of the farms: obviously not all farmers start from the same point, and they cannot all go at the same speed. The authorities will have to understand the need for gradual change: a progressive approach should be designed, with stages of compliance and timetables. It should allow those who want to go fast to do so while making sure to support all the farmers, regardless of their starting point.

Resources for the Regional Health Plan should be provided by ODARC through grants for farm restructuring. The TC made an initial estimate of the credits to be allocated. It has also reflected on the eligibility conditions for these subsidies and the commitments farmers would have to make to benefit from them. The aim is to ensure equal treatment between all the farmers.

The final step in this phase was the validation of the TC's work by the farmers' collective. The TC presented its results to stakeholders in three stages:

(i) consultation with the professional leaders of the four farmers' organizations;

(ii) design of a flier listing all the proposals, which was sent to all the farmers;

(iii) a regional debriefing meeting of the farmers' collective to present the work of the TC.

### Negotiating Acceptability With the Authorities

Once the farmers' collective had validated the TC's proposals, it was a question of meeting the expectations of the local officials of the state. Aware of the inadequacy of national measures with the Corsican context, the local officials of the state were ready to listen to the farmers' collective and TC proposals. They quickly accepted much of the reasoning behind the TC proposals and were ready to support the Regional Health Plan as a whole. These initial contacts between the farmers' collective (accompanied by the TC) and the local officials led to discussions about the proposals and the issues of biosecurity training, timelines, and controls (a requirement that the national authorities had imposed on the local officials). As a result, negotiations with national authorities were partially facilitated by the progressive enrollment of local officials to the Regional Health Plan.

The local officials clearly understood the broad scope of the Regional Health Plan, not only the issue of biosecurity on farms. In particular, there was intensive discussion of the issues of waste, hunting, slaughterhouses, and vehicle traffic between farms. The TC's work on these aspects provides a systemic vision of the issues connected with the dissemination of health hazards.

However, farm biosecurity remained a key element of the overall plan. The notion of pastureland and the hierarchy of risks between breeding stock and deli meat pigs soon arose in the discussions, along with the issues of oophorectomy and gestation control. However, the concept of the “leading sow” was the subject of in-depth reflection as the movement of these animals between protected and open areas can introduce significant risks.

The representativeness of the four farmers' organizations within the farmers' collective remained a sensitive point. Not all the identified positions were represented, and everyone was well-aware that the Plan was not spontaneously acceptable to all the farmers. But, the implementation of the Plan could isolate the recalcitrants, giving pledges to farmers willing to make efforts. Therefore, leaving the informality of a number of farmers while improving the structure of the sector as a whole became a medium-term objective of the Plan. So preparedness is an effective opportunity to stimulate the sector, and successful negotiation has major implications for solving the difficulties of a “problem” sector (slaughterhouses, trichinellosis, tuberculosis). This perspective makes it all the more important spending every effort to involve all parties, even those who resist.

Discussions with the national experts revealed another issue for negotiation: adaptations designed for one territory must not be available to all on the mainland. This was a condition the national authorities imposed to enable negotiations without losing control of the situation nationwide. In addition, the Regional Health Plan proposal includes the idea of taking specific measures to deal with a worrying zoonosis, bovine tuberculosis, with specific means and a timetable.

Finally, this phase complicated the acceptability testing insofar as differences of appreciation arose within the local officials and between them and the national authorities. Having a role for local specialist services in the decision chain has been essential for getting the authorities to understand the situation on the ground. In these discussions, the national experts played an important role in defining the acceptable and opening the way for local adaptations. So the process has involved a complex interplay between local experts and national experts on the one hand and local and national authorities on the other. The TC acts as a mediator in discussions to negotiate a solution (Figure 1).

This negotiation period is still ongoing at the time of writing, and it is still too early to know the final results.

## Discussion

### When Territorial Preparedness Meets “Local Universality”

In animal health management, the notion of “local universality” (40) is based on the idea that biosecurity measures are universal if they can be adapted to local contexts. It addresses the formal or informal negotiations made at the individual level that can make biosecurity measures practicable (41). However, when there is too great a gap between management principles built on a non-contextualized understanding of risk and locally specific configurations of a livestock sector, minor adaptations are not sufficient. This is especially true for the pig farming sector in Corsica, where a statistically marginal group includes the majority of farmers. Even local or minor adaptations of national biosecurity obligations would not have been acceptable to the farmers because its main thrust (keeping pigs off the open pasturelands) is in total contradiction with the Corsican systems.

The process of territorial preparedness is not a process of adaptation (in the sense of local universality) or a simple variation on a national measure according to a local context (42, in 11), but a bottom-up construction that creates new collective modes of pig health management at the level of a subnational territory. It transcends the usual barrier-based way of thinking about biosecurity measures (6). It is a collective construction that starts from a specific, territorial, multi-issue perspective (taking account of Corsican pig farming systems, proximity to Sardinia, insularity, the presence of other pig diseases, etc.) that does not rely only on the epidemiological point of view (5). In a sense, as territorial preparedness fosters a “bottom-up perspective” encompassing specific territorial configurations, it encounters the “top-down” perspective of local universality, stressing the creative capacities of local stakeholders, including the regional health administration.

The outcome of the process is not yet known, but it could represent an “[…] *autonomous system of collective action […], empowered by its specific modes of governance in accordance with local values and behaviors*” (Dubresson and Jaglin, 2005, 11)—a shift to genuinely subnational risk management. If the plan is accepted, the island of Corsica cannot be considered an “area at risk” or a “sentinel island” from an epidemiological point of view (21) because of “particular ways” of breeding pigs that pose a particularly high risk of disease spillover. Instead, it would be a “risk-prepared territory” (11), thanks to the reframing of the risk in a “*singular, situated, and dated relation to a society and a territory*” (11). So, as this territorial preparedness takes into account the specific technical features of the Corsican pig farming systems and the epidemiological risk of ASF, the French government is likely to accept this alternative. There are two final arguments in favor of such acceptance. First, the fact that Corsica is an island limits the risk of ASF spillover to mainland France. Second, by acknowledging Corsica's particularities, the state authorities can strictly limit the proposed biosecurity norms to this territory without opening the door to administrative divisions on the mainland where similar difficulties may occur.

### Finding the Way Toward Compromise: The Role of Local Expertise in the Mediation Process

Our results show several steps of acceptability testing in the process of building subnational territorial preparedness mediated through the work of the TC. Mediation process is crucial for territorial preparedness at subnational levels. The negotiations were not between two coherent groups (government vs. farmers). The pig sector includes a wide diversity of farming systems (and a diversity of positions), and the public authorities also are not homogeneous. Public servants in the region played a crucial role in technical discussions and the negotiation round with the national authorities. The diverse nature of the public authorities in terms of local anchorage and practices is an important point from a public management point of view (43, 44) for any further investigation of the dynamics of building territorial preparedness.

The preparedness reasoning was carried out by “local expertise,” and government experts opened a space for discussion of technical and organizational issues in participative settings. This eased the potential political tensions such as those that often emerge when government policy is implemented in a top-down manner (45). Taking part in essentially technical and organizational discussions, the researchers and development officials avoided adopting advocacy positions. Being both internal actors (as experts) and external actors (drawing no benefit from the results of the negotiation), they avoided, in analysis and interpretation of results, the possible biases such as those that have been highlighted in previous research on participative approaches (46, 47). The best reflection of this is the credibility that the State representatives accorded the proposals. The involvement of public sector researchers can help small, poorly organized farming systems on the margins of mainstream agricultural production by providing a discussion space for exploring their prospects.

One illustration of this is the “traditional” aspect of Corsica's pig sector. As a major justification for the PDO application, the traditional practices shaped the requirements linking the way the pigs are herded with the typicality of the deli meat products. However, in the early discussions, tradition was used (particularly by the “passive” farmers) as an argument for rejecting any change imposed by the national biosecurity plan. This argument was refuted by old breeding control practices: traditionally, hybridization was prevented by locking up the sows during the mating season (48). The current, recently adopted practices (lack of breeding control in full free-range) cannot be presented as “traditional” (even if the “passive” farmers do so). The proposed changes for an adapted biosecurity may be seen as a return to the real tradition in which domestic pigs and wild boars were strictly separated in the mating season. In fact, “tradition” is an interpretation of the past from the standpoint of the interests and opinions of the present (49).

As a result, local farming systems gained legitimacy not on a “heritage” basis (the supposed tradition) but on the local stakeholders' commitment to transform the pig sector in order to improve general biosecurity and preparedness. This shows a kind of paradox, in that preserving Corsica's pig farming systems depends on changing them. This “normalization” is acceptable to the farmers because it preserves their mode of production (use of pasturelands) and the associated benefits (government subsidies) and offers them the possibility of negotiating for additional subsidies to enable farmers to reach the collectively established norms. Under these conditions, Corsican farmers get guarantees from the state, unlike other places such as Sardinia, where the implementation of biosecurity standards drove free-range systems to extinction by declaring them illegal (50). Without the mediated process, there was likely to be widespread rejection of the national biosecurity plan, with many farmers joining the informal sector. The mediation process made it possible to develop such considerations and get them acknowledged, facilitating learning and building trust through shared consideration of each participant's issues (27). Judging by our experience with small farmers' issues, such trust building is a key condition for success in collective action.

### The End of an Embarrassing Notion: Toward Democratic Acceptability

When social acceptability is treated as a dynamic process, it is no longer an “embarrassing notion” (26, 28) that would imply getting local actors to accept measures they do not want. The territorialized preparedness process is the result of a collective bottom-up dynamic that legitimizes not only the biosecurity co-construction process but also the actors who carried it out. At the local level, the farmers' collective, representing the interests of all the farmers, and the TC are emerging as recognized actors in health management. They transcend the old divisions between individual farmers and between organizations (each with its own economic or socio-technical objectives) and outline a new form of collective action for health that the existing farmers' organizations did not provide (51). We observed here the start of a completely new form of consultation within the sector: all the pig farmers united against a common danger. The collective dimension of animal health management is thus affirmed. The authorities—both national and local—now recognize the TC and the farmers' collective as negotiating partners and acknowledge the preparedness-building process and its output, the Regional Health Plan, which could lead to subnational-level health governance (52).

The legitimacy of the actors involved in the social acceptability process is central (27). The legitimacy of the TC was built up during the process, with each member of the TC earning recognition within the TC and the TC becoming legitimate in the eyes of both the state and the farmers, specifically through its collective local expertise, but also because it brought together all the actors connected with livestock health. This approach to preparedness, built on a process-based, bottom-up, regionally differentiated mode ((53), in 26), makes traditional Corsican farming systems acceptable, despite being unacceptable from the standpoint of national biosecurity standards. Moreover, the whole process, through technical and organizational discussions, has made biosecurity and stakeholder issues on the island more visible and comprehensible. It helped to build trust between stakeholders, TC members, the authorities, and farmers, so that they could pursue the co-production process (27).

The acceptability of territorial preparedness is a democratic process. On the one hand, Corsican farmers and other stakeholders have become actors in their own future (26); on the other hand, the state has agreed to negotiate with a marginal region that views norms in light of its own issues. In so doing, it recognizes as legitimate the extensive farming systems. In terms of biosecurity and risk management, the real problem is not the farming system but the way in which the territory and its stakeholders are involved in decisions about management measures. Marginal systems can be regarded as threats to the proper functioning of society or they can be a hotbed of innovation, fostering democratic experimentation (54). If the government accepts Corsica's preparedness project, this will probably be due to the fact that both the territory and the farming system are marginal. The democratic experiment will be more easily accepted in the Corsican case because it will remain circumscribed to this island territory, a condition set by the state from the outset.

This collective experiment in co-production of an acceptable ASF preparedness specific to the island highlights the emergence of a “style of reasoning” about preparedness (4). By including stakeholders' particular issues and representations and generating local legitimacy, it may avoid the programmed failure of national guidelines, procedures, and instruments (4, 10) in animal disease prevention.

For One Health or planetary health to be perhaps more effective and operationalizable, it seems important not to stigmatize marginal areas and alternative farming systems by forcing change on them. Instead, there should be coordination to build the conditions for biosecurity measures that farmers can accept. A striking counter-example is Sardinia, where outlawed extensive farming is largely responsible for the failure of a number of eradication plans. After a 40-year struggle against ASF, a coordination of various local experts, the *Unita di progetto* (55), has recently been formed and seems to be producing good results. This example and the Corsican preparedness plan are strong arguments for integrated, coordinated, locally oriented approaches to emerging diseases rather than standardized top-down approaches.

## Conclusion

This article has described and analyzed the building of an alternative preparedness solution to fight against ASF by means of action research conducted by the authors of this article.

First, we demonstrate the unacceptability of the national measures from the viewpoint of the farmers concerned and formalize the arguments that forged it. We explain and analyze their arguments and the various positions they reveal. The clash between these arguments led to the creation of a new body, the regional TC. Then, through a long iterative process, the TC developed a Regional Health Plan that takes into account the specific features of the smallholder farmers' situation and incorporates the need to protect them from health hazards, not only the emerging one of ASF but also those that are already present. The TC first submitted the proposed Plan to the smallholder farmers and their organizations because many of these smallholders are not prepared to make major changes to their farming practices. Finally, this Regional Health Plan, which includes biosecurity measures specific to local farms, became the subject of negotiations with the authorities.

The process is original in several respects. (i) The TC did not simply seek to adapt the national biosecurity standards to Corsican farms. It constructed new proposals that constitute overall ASF preparedness for a specific territory. This locally specific preparedness plan engages the responsibility of a multiplicity of actors, not only the farmers, and addresses not only the ASF risk but also health hazards already present. Biosecurity and the husbandry measures were designed to preserve the use of the free range while avoiding interactions with wildlife. (ii) The TC, as a committee of experts, not only made proposals but also acted as intermediary between the farmers and the state, making sure the proposals were acceptable first to the farmers and then to the authorities. The TC organized the conditions that made negotiation possible. (iii) The collective action initiated by the TC seems to inaugurate a new way of thinking about the governance of animal health in Corsica. More generally, the crucial role played by the TC in the process offers useful ideas about empowering public action through local mediation and co-production capacities, especially in France, where animal health matters are centrally governed.

The process of territorial preparedness in Corsica shows that there are alternatives to standardized biosecurity and the risk of disappearance of small farmers and their traditional systems. The legitimacy of these farms should be established in partnership with other local stakeholders through a regional approach to risk and bottom-up construction of preparedness.

## Data Availability Statement

The raw data supporting the conclusions of this article will be made available by the authors, without undue reservation.

## Ethics Statement

Ethical approval was not provided for this study on human participants because an oral agreement is established on the researchers role in such collective process. Participants of this study were informed in advance about details of how the data would be used, assuring anonymity, and informed consent was orally obtained. Written informed consent for participation was not required for this study in accordance with the national legislation and the institutional requirements.

## Author Contributions

MG and FCa contributed to the initiation and supervision of research. MG, FCa, and BT participated in the action-research process and data production. MG, FCa, FCh, and BT contributed to the conception, data analysis, visualization and writing, and manuscript revision. All authors contributed to the article and approved the submitted version.

## Conflict of Interest

The authors declare that the research was conducted in the absence of any commercial or financial relationships that could be construed as a potential conflict of interest.

## Publisher's Note

All claims expressed in this article are solely those of the authors and do not necessarily represent those of their affiliated organizations, or those of the publisher, the editors and the reviewers. Any product that may be evaluated in this article, or claim that may be made by its manufacturer, is not guaranteed or endorsed by the publisher.
